# Efficacy of ARV-1502, a Proline-Rich Antimicrobial Peptide, in a Murine Model of Bacteremia Caused by Multi-Drug Resistant (MDR) *Acinetobacter baumannii*

**DOI:** 10.3390/molecules24152820

**Published:** 2019-08-02

**Authors:** Yan Q. Xiong, Liang Li, Yufeng Zhou, Carl N. Kraus

**Affiliations:** 1 Los Angeles Biomedical Research Institute at Harbor-UCLA Medical Center, Torrance, CA 90502, USA; 2 Geffen Sch. of Med. at UCLA, Los Angeles, CA 90095, USA; 3 Arrevus, Inc. Research Triangle Park, NC 27612, USA

**Keywords:** multi-drug resistant *Acinetobacter baumannii*, antimicrobial peptide, bacteremia, host defense peptide

## Abstract

*Acinetobacter baumannii* bacteremia represents a serious and increasing clinical problem due to the high mortality and treatment failures because of high rates of antibiotic resistance. Any additional new therapies for *A. baumannii* bacteremia would address a growing unmet medical need. ARV-1502 (designated as Chex1-Arg20 or A3-APO monomer in prior publications) is a designer proline-rich antimicrobial peptide chaperone protein inhibitor derived from insects and has demonstrated potent activity against multi-drug resistant (MDR) Gram-negative bacteria. In the current studies, we investigated the therapeutic efficacy of ARV-1502 administered intravenously (iv) alone and in combination with imipenem/cilastatin (IPM/CIL) in a mouse bacteremia model due to a MDR clinical *A. baumannii* strain, HUMC1. All ARV-1502 regimens (1.25, 2.5 and 5.0 mg/kg) significantly reduced bacterial density in the target tissues in a dose-dependent manner, as compared to the untreated control and IPM/CIL monotherapy (40 mg/kg) groups in the model. In addition, ARV-1502 treatment, even at the lowest dose, significantly improved survival vs. the control and IPM alone groups. As expected, IMP/CIL monotherapy had no therapeutic efficacy in the model, since the HUMC1 strain was resistant to IMP in vitro. However, the combination of ARV-1502 and IPM/CIL significantly enhanced the efficacy of ARV-1502, except the lowest dose of ARV-1502. The superior efficacy of ARV-1502 in the bacteremia model caused by MDR *A. baumannii* provides further support for studying this compound in severe infections caused by other MDR Gram-positive and -negative pathogens.

## 1. Introduction

Bloodstream infections (BSIs) caused by multi-drug resistant (MDR) bacterial pathogens are becoming more prevalent and are often life-threatening, with significant morbidity and mortality primarily due to the antibiotic-resistant nature of the pathogens [1]. These MDR pathogens, including *Acinetobacter baumannii*, have been declared serious threats to public health by the centers for Disease Control and Prevention [2]. Treatment is typically empiric for patients at high risk of harboring MDR Gram-negative pathogens, and options are limited to anti-pseudomonal β-lactams, carbapenems in combination with a quinolone or aminoglycoside. Imipenem (IPM), a standard-of-care β-lactam antibiotic, has a broad spectrum of activity against aerobic and anaerobic, Gram-positive and -negative bacteria, including many MDR strains (e.g., *A. baumannii*). However, many *A. baumannii* strains have developed resistance to IPM. Thus, the lack of effective treatment options presents an urgent need for developing antimicrobial agents with novel mechanism of action.

Antimicrobial peptides, a growing class of natural and synthetic peptides, have been considered powerful drugs against a broad spectrum of pathogens, including those that are resistant to conventional antibiotics. However, the in vivo therapeutic efficacy of these peptides is occasionally hampered by inadequate safety margins and frequently by rapid clearance, leaving them suitable only for topical applications [3,4].

Arrevus is developing a unique class of antimicrobial peptides derived from insects that act via a novel mode of action against infections caused by MDR Gram-negative bacteria. Specifically, peptide sequences from insect antimicrobial peptides, such as drosocin (*Drosophila melanogaster*) and pyrrhocoricin (*Pyrrohocoris apterus*), were used to engineer consensus sequences for activity screening assays, and DnaK was determined to be a target [5]. DnaK is a 650-residue protein comprised of three distinct domains: (1) N-terminal nucleotide binding domain, (2) C-terminal substrate binding domain and (3) flexible linking domain [6]. ARV-1502 binds to DnaK with a K_d_ of 0.41 (± 0.01) μM [7], with in vitro activity against multiple Gram-negative bacteria demonstrating antibacterial potency [8]. Binding of ARV-1502 to DnaK inhibits opening and closing of the multi-helical lid over the peptide-binding pocket of DnaK and the legacy pyrrhocoricin sequence has been shown to inhibit the DnaK-mediated phosphate release from ATP [9]. ARV-1502 binds to DnaK from multiple bacterial species and the membrane permeabilizing concentration (MPC) was noted to be > 100 μM, supporting its activity as a non-membrane lytic peptide [10].

This approach exploits Arrevus’ antimicrobial peptides ARV-1502 which inhibit critical bacterial proteins, responsible for bacterial protein folding, such as chaperone proteins. Designer proline-rich antimicrobial peptides act as chaperone protein inhibitors (DPCs), which are key elements of insects’ innate immune response [11,12,13,14,15]. The DPCs have been engineered to maximize their activity as anti-infective, with some incorporated sequences promoting membrane penetration and inhibiting DnaK (pyrrhocoricin from *P. apterus*). Arrevus’ lead DPC, ARV-1502 (also named as Chex1-Arg20 amide in previous publications [16,17]), a monomeric metabolite of ARV-1501 (also called as A3-APO), represents a novel strategy specifically targeting bacterial DnaK, resulting in the bacteria’s inability to overcome an antibiotic challenge for treating bacterial infections, and enhancing companion antibiotic activity against MDR bacteria. The current study was designed to test the in vitro activity and in vivo efficacy of intravenous ARV-1502 administration, used alone or in combination with a FDA-approved antibiotic, imipenem/cilastatin (IPM/CIL) that is indicated for bacteremia, in a bacteremia model of MDR *A. baumanii*.

## 2. Results

### 2.1. In Vitro Susceptibility of ARV-1502 Alone and in Combination with IPM

The minimum inhibitory concentration (MIC) of ARV-1502 was 50 μg/ml against the *A. baumannii* strain, HUMC1. This strain was resistant to IPM (MIC = 16 μg/ml). Importantly, ARV-1502 had additive activity in combination with IPM against the MDR *A. baumannii* strain. 

### 2.2. Therapeutic Efficacy of ARV-1502 Alone and in Combination with IPM/CIL in a Murine Model of Bacteremia due to the MDR A. Baumannii Strain HUMC1

**Figure 1:**
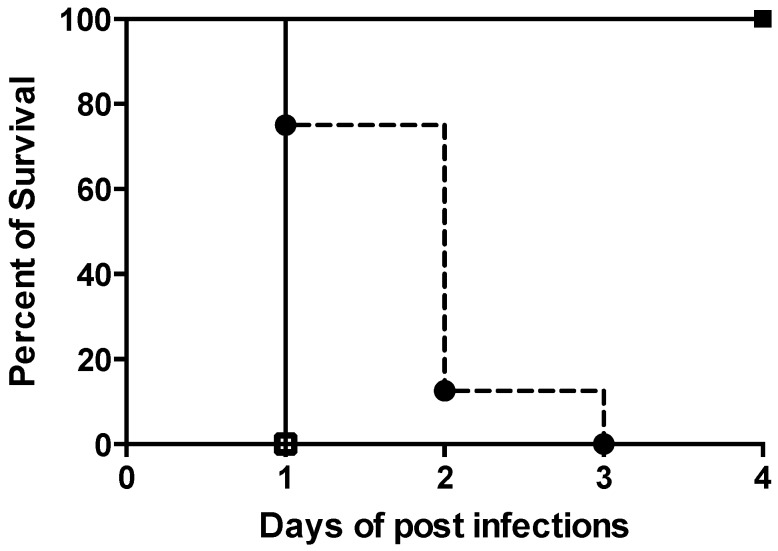
Percent of survival of *A. baumannii*-infected mice with 10^9^ CFU/mouse (□) 10^8^ CFU/mouse (●) or 10^7^ CFU/mouse (■).

We first demonstrated 100% mortality in animals infected intravenously (iv) with 10^9^ CFU/mouse of the *A. baumannii* strain at 24 h post-infection (Figure 1). In addition, 100% mortality was observed in animals infected by 10^8^ CFU/mouse of the *A. baumannii* strain at 3 days post-infection (Figure 1). 

Bacterial densities in kidneys were similar between 10^8^ and 10^9^ CFU/mouse infection doses but were significantly higher in spleen and liver in animals infected by 10^9^ CFU vs. 10^8^ CFU/mouse (Table 1). Moreover, all mice infected with 10^7^ CFU/mouse survived at 4 days post-infection (Figure 1), with ≥50% negative cultures in the target tissues (kidney, spleen and liver; Table 1). Thus, an ID_95_ dose of 10^8^ CFU/mouse was used for the efficacy experiments. 

**Figure 2:**
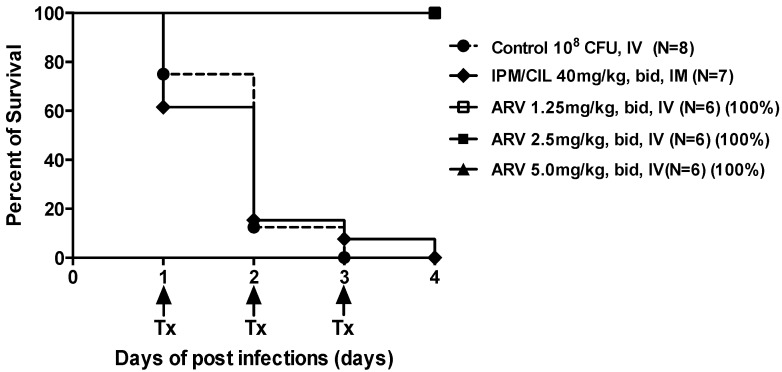
Survival of *A. baumannii*-infected mice with a defined ID_95_ (2 × 10^8^ CFU/animal) following no treatment (control), treatment with ARV-1502, imipenem/cilastatin (IPM/CIL) alone or in combination. Tx: treatment.

All ARV-1502 monotherapies, including 1.25, 2.5 and 5.0 mg/kg, had 100% survival, while untreated control and IPM/CIL alone treated animals had 100% mortality at 4 days post-infection (Figure 2). 

**Figure 3:**
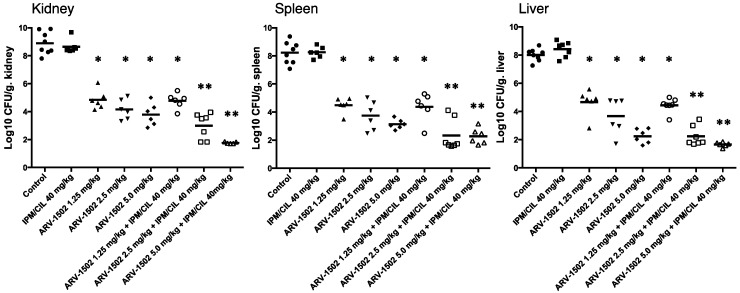
*A. baumannii* burden in the target tissues (kidney, spleen and liver) were quantified as CFU per gram of target tissues in the murine bacteremia model following different regimens. * *p* < 0.001 vs. control and IPM/CIL alone groups, ** *p* < 0.05 vs. respective ARV-1502 monotherapy group, using Student’s *t*-test. At least six mice per group were analyzed.

Also, all the ARV-1502 treatment groups significantly reduced *A. baumannii* densities in the target tissues in a dose-dependent manner vs. untreated control and IPM/CIL alone treated groups (Figure 3). As expected, in the model, when bacteremia is established IPM/CIL alone failed to impact survival and bacterial density in any of the target tissues as compared to the control group (Figure 2 and Figure 3 for survival and bacterial counts, respectively). 

**Figure 4:**
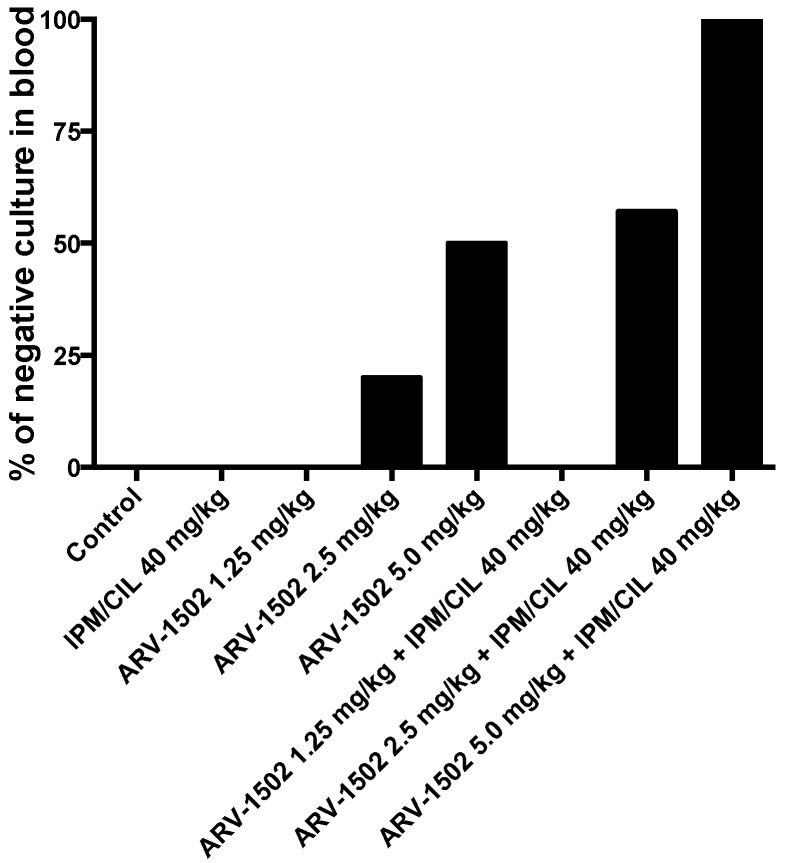
Percent of negative cultures in blood in the murine bacteremia model following different regimens.

Interestingly, the combination of ARV-1502 with IPM/CIL significantly enhanced the therapeutic efficacy of ARV-1502 alone, except at the lowest dose of ARV-1502 (1.25 mg/kg, Figure 3). Additionally, the combination of ARV-1502 (at 1.25, 2.5 or 5.0 mg/kg) with IPM/CIL also resulted in 100% survival, which is the same as the ARV-1502 monotherapy groups. Moreover, a dose-dependent manner of ARV-1502 with negative blood culture of *A. baumannii* was observed (Figure 4). These data suggest that ARV-1502 at 1.25, 2.5 and 5.0 mg/kg iv, bid for 3 days had potent anti-MDR *A. baumannii* activity in the murine bacteremia model. 

## 3. Discussion

It has been reported that ARV-1502 is active against a broad range of MDR Gram-negative bacterial species, including *A. baumannii, P. aeruginosa, E. coli,* and *K. pneumoniae* [18,19]. For instance, the peptide showed inhibitory activity on the growth of *E. coli* and *K. pneumoniae* strains with MIC values of 6 and 2 μg/ml, respectively [17,20]. In addition, in combination with a traditional antibiotic (e.g., IPM), ARV-1502 has the potential to enhance that activity of this antibiotic against the MDR *A. baumannii* strain. Since prior work has demonstrated a high binding affinity of ARV-1502 for *E. coli* DnaK [7], the increased anti-MDR bacteria activity of ARV-1502 in combination with the antibiotic might be at least in part due to the impact of the antibiotics on bacterial membrane integrity, which improves the penetration of ARV-1502 across the bacterial membrane and enhances its activity.

The current studies demonstrated that ARV-1502 via an iv route at all tested doses (1.25, 2.5 and 5.0 mg/kg) significantly enhanced the survival of mice in the experimental bacteremia model due to MDR *A. baumannii* (0 and 100% survival for untreated control and ARV-1502 treated groups, respectively). In addition, ARV-1502 significantly reduced bacterial load in the target tissues in a dose-dependent manner vs. untreated control and IPM/CIL monotherapy groups. Supporting our data, previous studies showed that proline-rich antimicrobial peptides, as monotherapy, had therapeutic efficacy against a *K. pneumonia* murine thigh infection model and an *E. coli* mouse peritonitis model [21,22]. As expected, IPM/CIL treatment alone did not have therapeutic efficacy in the bacteremia model. However, in the combination treatment studies, ARV-1502 significantly enhanced the activity of IPM/CIL, as demonstrated by the improvement of survival, as well as reduction of *A. baumannii* load in the target tissues. Importantly, ARV-1502 enhanced the survival of mice in the MDR *A. baumannii* bacteremia model without noticed systemic toxicity. 

In the current study, the studied MDR *A. baumannii* strain had a MIC value of 50 μg/ml, which could be considered resistant to ARV-1502 in vitro, but the strain was remarkable susceptible to ARV-1502 treatment, even at the lowest dose, in the experimental bacteremia model. Consistent with our findings, other proline-rich antimicrobial peptides have been reported to have great efficacy against *P. acnes*, *A. baumannii* and *S. aureus* in systemic infection models, in spite of high MIC values against these pathogens [18,23,24,25]. These data support the historically unreliable use of MIC data as a predictor of in vivo activity of antimicrobial peptides, and, suggests that the mode of action of such peptides is not limited to direct bacterial killing. Rather, there is likely activation of host defense mechanisms as an additional mode of action [18]. Growing evidences from many studies with relevant infection models have supported this notion, indicating that the host immune system can be activated by external antimicrobial peptides, which is equally, or even more important than their antimicrobial activity. For instance, Ostorhazi et al. recently reported that ARV-1502 treated mice induced >5-fold production of anti-inflammatory cytokine IL-10 vs. untreated animals [19]. These data suggest that the improved in vivo efficacy of ARV-1502 is likely associated with increased host immune system upon infection [19]. 

Like other antibiotic approaches, antimicrobial peptides have displayed reduced therapeutic utility due to several reasons. First, bacteria are able to develop resistance by neutralizing antimicrobial agents through a variety of methods, including mutations of genes related to decreased membrane charges [19,26]. It has been demonstrated that the Arrevus’s insect-derived antimicrobial peptides, functioning not as typical membrane-disruptive peptides, have the potential to address such antibiotic resistance concerns [16,17,19]. For instance, previous studies have shown that resistance to ARV-1502 was not seen for the duration of 35 in vitro passages, which might due to its unique mechanism of action against a ubiquitous and highly conserved bacterial protein [12]. These results suggest that resistance to ARV-1502 is less likely than resistance to standard of care antibiotics. 

In summary, these exciting in vivo results have provided significant support for further investigations of ARV-1502 alone and in combination with standard-of-care antibiotics to manage MDR bacterial infections. 

## 4. Materials and Methods 

### 4.1. Bacterial Strain and Growth Conditions

A MDR and highly virulent clinical isolate of *A. baumannii* (HUMC1) was used. This strain was isolated from the blood and sputum of a patient seen at Harbor-UCLA Medical Center (Torrance, CA) and obtained from the Clinical Microbiology Laboratory at the Medical Center. It is resistant to all clinically useful antibiotics reported by the clinical microbiology laboratory, except for colistin, and has been found to be hypervirulent in experimental murine models of infection [27,28]. The HUMC1 strain was cultured using aseptic technique and routinely grown in tryptic soy broth (TSB) or on tryptic soy agar (TSA) plates. 

### 4.2. Molecular Design and Synthesis of ARV-1502

ARV-1502 is an engineered non-membrane disruptive antimicrobial peptide that was developed as a consensus sequence from known native proline-rich antibacterial peptides that, as a class, carry non-lytic mechanisms of action (membrane permeabilization concentration, MPC > 100 μM). This peptide was synthesized and purified by reversed-phase high-performance liquid chromatography (RP-HPLC) as previously described [19]; purity was 96.8%. The actual peptide content of ARV-1502 was quantified by amino acid analysis or RP-HPLC and published previously (H-Chex-Arg-Pro-Asp-Lys-Pro-Arg-Pro-Tyr-Leu-Pro-Arg-Pro-Arg-Pro-Pro-Arg-Pro-Val-Arg-NH2). In addition, ARV-1502 is a monomeric metabolite of ARV-1501, which has been extensively studied [24,29]. 

### 4.3. Determination of MICs

The MICs of ARV-1502 and IPM were tested by a standard broth microdilution assay using 96-well plates as recommended by the Clinical and Laboratory Standards Institute [30]. The MICs were defined as the lowest antimicrobial agent concentrations when turbidity was not observed.

### 4.4. Fractional Inhibitory Concentration Index (FICI) Assay

Conventional checkerboard assays were performed with ARV-1502 in combination with IPM against the MDR *A. baumannii* HUMC1 strain in order to identify combinational activity according to standard protocols [31]. ARV-1502 was serially diluted 1:1 to provide an 11-point concentration range in a volume of 50 µL. The standard antibiotics (imipenem, colistin, tigecycline and piperacillin) were serially diluted 1:1 to provide a 7-point concentration range in a volume of 50 µL. To each well containing ARV-1502 and antibiotic, approximately 5 × 10^5^ CFU of bacteria were added and incubated for 24 hours in an ambient atmosphere at 37 °C. After 24 hours, the presence of turbidity was assessed and MIC values of both test article and antibiotic, as well as the fractional inhibitory concentration index (FICI) values were assessed. Concentrations for additive activity were noted to be 3.13 and 0.50 μg/mL for imipenem and ARV-1502, respectively. The FICI was determined for each combination with <0.5 indicating synergy, 0.5–4 indicating addition, and >4 indicating antagonism [31]. The FICI assays were performed in triplicate to ensure repeatability and characterize assay variability. 

### 4.5. ID_95_ in a Murine Bacteremia Model due to A. Baumannii (HUMC1) 

Prior to the efficacy studies, we defined an ID_95_ in the experimental mouse bacteremia model. For these studies, CD-1 mice (18–23 g, female) were infected intravenously (iv) with log-phase cells (~3 hr incubation) of the *A. baumannii* strain, HUMC1 at 10^7^, 10^8^ or 10^9^ CFU/mouse inoculum. The percent of survival during the 4 days post-infection were calculated. Bacterial densities in target tissues (e.g., kidney, spleen, liver) were quantified by serial dilution on TSA plates and expressed as log_10_ CFU/g. tissues.

### 4.6. Efficacy of ARV-1502 and IPM/CIL Alone, and in Combination in A. Baumannii (HUMC1)—Challenged Murine Bacteremia

For the efficacy experiments, animals were infected iv at the ID_95_ (10^8^ CFU/mouse) of the *A. baumannii* strain defined above. At 24 h post-infection, animals were randomized to receive one of the following regimens (6–8 animals/group): (i) Untreated controls; (ii) Imipenem/cilastatin (IPM/CIL) alone at 40 mg/kg, intramuscular (im), bid; (iii–v) ARV-1502 at 1.25, 2.5 or 5.0 mg/kg, iv, bid; or (vi–viii) ARV-1502 at 1.25, 2.5 or 5.0 mg/kg, iv, bid in combination with IPM/CIL at 40 mg/kg, im, bid. Treatment lasted for three days. Since IPM is rapidly degraded by renal enzyme dehydropeptidase 1 when administered alone, it is co-administered with CIL to prevent this inactivation [32]. At 24 h after the last therapeutic dose, animals were sacrificed and target tissues (e.g., kidney, spleen and liver) were removed, weighed, homogenized, serial diluted in PBS and quantitatively cultured. Bacterial counts in the target tissues were calculated from each group and expressed as mean (± SD) log_10_ CFU/g. tissues or log_10_ CFU/ml of blood. 

Animals were maintained and handled in accordance with the recommendations of the Guidelines for the Care and Use of Laboratory Animals, and the protocols were approved by the Animal Care Committee of Los Angeles Biomedical Research Institute at Harbor-UCLA Medical Center. 

### 4.7. Statistical Analysis

To compare tissue *A. baumanii* counts in the various groups, unpaired Student’s *t*-test was used. *p* values of <0.05 were considered statistically significant.

## Figures and Tables

**Table 1:** ID_95_ studies in a murine bacteremia model due to multi-drug resistant (MDR) *A. baumannii* strain, HUMC1.

Infection Doses (No. Animals)	Mean log_10_ CFU/g. Tissue ± SD (% Sterile Culture)		
	Kidney	Spleen	Liver
10^9^ CFU (*n* = 6)	8.71 ± 0.21 (0%)	9.71 ± 0.31 (0%)	9.33 ± 0.21 (0%)
10^8^ CFU (*n* = 8)	8.90 ± 0.80 (0%)	8.22 ± 0.79 (0%)	8.01 ± 0.44 (0%)
10^7^ CFU (*n* = 6)	2.41 ± 0.82 (50%)	1.86 ± 0.15 (100%)	2.08 ± 0.39 (66.7%)
